# Panoramic radiograph–based dental age estimation in children and adolescents using multi-tooth methods: a scoping review of methods used and reported accuracy (2020–2025)

**DOI:** 10.1186/s12903-026-09263-w

**Published:** 2026-07-15

**Authors:** Małgorzata Łysiak-Majchrzak, Sylwia Jagła, Natalia Kazimierczak, Wojciech Kazimierczak, Teresa Matthews-Brzozowska

**Affiliations:** 1 Department of Maxillofacial Orthopedics and Orthodontics, Ludwik Rydygier Collegium Medicum in Bydgoszcz, Nicolaus Copernicus University in Toruń, Bydgoszcz, Poland; 2 Kazimierczak Clinic, Dworcowa 13/u6a, Bydgoszcz, Poland; 3 Department of Radiology and Diagnostic Imaging, Ludwik Rydygier Collegium Medicum in Bydgoszcz, Nicolaus Copernicus University in Toruń, Bydgoszcz, Poland

**Keywords:** Dental Age, Child, Tooth, Willems Method, Cameriere Method, Accuracy, Panoramic radiography

## Abstract

**Background:**

Accurate dental age estimation supports treatment planning in pediatric dentistry and orthodontics and forensic assessment when chronological age is uncertain. Because the accuracy of panoramic radiograph-based methods varies across populations, age groups, and method variants, a contemporary synthesis is needed to guide method selection.

**Objective:**

To map the methods used for panoramic radiograph–based dental age estimation in children and adolescents and to summarize the reported diagnostic accuracy of the most frequently used method families in studies published between 2020 and 2025.

**Methods:**

This scoping review was conducted using PubMed, Scopus, and Web of Science. Studies published between 1 January 2020 and 31 December 2025 were screened according to predefined eligibility criteria. Data were extracted using a standardized form, including study characteristics, dental age estimation methods, and reported accuracy outcomes (primarily mean absolute error [MAE] and mean difference/bias [DA–CA]). Thirty-four original studies were included.

**Results:**

The included studies comprised over 35,000 children and adolescents from multiple countries and were predominantly retrospective and clinic-based. Willems-family methods were the most frequently reported, followed by Demirjian-, Cameriere-, and London Atlas-based approaches. Across studies, accuracy reporting was heterogeneous with respect to MAE, bias metrics, sex stratification, and method variants. Cameriere family methods often showed lower MAE values in many cohorts; however, performance varied by population, age range, and method modification.

**Conclusion:**

Panoramic radiograph–based dental age estimation methods show substantial heterogeneity in use and reported accuracy across populations. Willems-family methods are the most frequently applied, whereas Cameriere-family methods often demonstrate favorable accuracy in many cohorts, but no single method can be considered universally optimal. Population-specific validation and standardized reporting of accuracy metrics are needed.

**Supplementary Information:**

The online version contains supplementary material available at https://doi.org/10.1186/s12903-026-09263-w.

## Introduction

Instrumental assessment of dental age in children is primarily based on two imaging modalities: panoramic radiography and cone-beam computed tomography (CBCT). Panoramic radiographs are widely used in pediatric dentistry as a routine diagnostic tool. Their popularity stems from the ability to visualize the entire dentition in a single image while maintaining a relatively low radiation dose, particularly with modern digital systems that significantly reduce exposure compared with earlier analog techniques. Panoramic radiographic methods that enable the assessment of tooth germ mineralization constitute a safe and effective approach to evaluating dental development in children and adolescents [1].

Accurate dental age estimation is clinically relevant across several disciplines. In paediatric dentistry, it supports the timing of preventive interventions, monitoring of eruption sequences, and identification of developmental anomalies such as delayed or accelerated dental maturation [2]. In orthodontics and dentofacial orthopaedics, knowledge of the developmental stage is integral to treatment planning decisions, including the optimal timing for functional appliance therapy, growth modification, and the initiation of fixed appliance treatment [3, 4]. In paediatric oral surgery, dental age informs decisions regarding the extraction of primary teeth and the management of impacted or supernumerary teeth [2]. Beyond direct clinical care, dental age estimation has established forensic applications in the identification of unaccompanied minors and age-disputed individuals in legal and humanitarian contexts, where no documentary evidence of birth date is available [5–7]. Given the breadth of these applications and the diversity of populations served by dental clinicians worldwide, the selection of an appropriate and validated dental age estimation method is a matter of practical clinical importance.

Over the years, numerous methods for dental age estimation have been developed and refined, including approaches proposed by Demirjian, Willems, Cameriere, Nolla, Haavikko, Moorrees, the London Atlas, and others [8–11]. The popularity of individual dental age estimation methods has varied over time and across different regions of the world, reflecting local preferences, population-specific validations, and the availability of reference data.

The aim of the present scoping review was to map contemporary panoramic radiograph–based dental age estimation methods used in children and adolescents and to summarize their reported diagnostic accuracy in studies published over the past five years.

## Materials and methods

### Search strategy and information sources

The review question was structured using the Population–Concept–Context (PCC) framework, which is commonly applied in scoping reviews [12]. The Population comprised children and adolescents. The Concept encompassed multi-tooth dental age estimation methods based on panoramic radiographs and the reported accuracy of these methods. The Context comprised clinical and forensic dental age-estimation research across diverse populations and geographic settings. The 2020-2025 publication period was an eligibility criterion, not a PCC component. The review question was: Which multi-tooth panoramic radiograph–based methods are used to estimate dental age in children and adolescents, in recent literature, and how is their accuracy reported?

This scoping review was conducted and reported in accordance with the PRISMA Extension for Scoping Reviews (PRISMA-ScR) [12]. The literature search was performed in three eletronic databases: PubMed, Scopus and Web of Science. No formal review protocol was registered or published before conducting this scoping review. Searches targeted studies published between 1 January 2020 and 31 December 2025; the final search was performed on 31 December 2025. No database filters were applied. Eligibility was restricted to full-text articles published in English to enable consistent extraction and interpretation of methodological and accuracy data. Backward and forward citation chasing was not performed.

The complete database-specific search strategies were as follows:

- PubMed: ((“dental age“[tiab] OR “dental maturation“[tiab] OR “dental maturity“[tiab] OR “dental development“[tiab] OR “age estimation“[tiab]) AND (radiographic[tiab] OR radiological[tiab] OR “panoramic radiograph“[tiab] OR panoramic[tiab] OR “orthopantomogram“[tiab] OR OPG[tiab] OR “X-ray“[tiab] OR xray[tiab] OR “x ray“[tiab]))
- Scopus: TITLE-ABS-KEY ( (“dental age” OR “dental maturation” OR “dental maturity” OR “dental development” OR “age estimation”) AND (radiographic OR radiological OR “panoramic radiograph” OR panoramic OR orthopantomogram OR OPG OR “x-ray” OR xray OR “x ray”) )
- Web of Science: TS=((“dental age” OR “dental maturation” OR “dental maturity” OR “dental development” OR “age estimation”) AND (radiographic OR radiological OR “panoramic radiograph” OR panoramic OR orthopantomogram OR OPG OR “x-ray” OR xray OR “x ray”))

All retrieved records were imported into Zotero and deduplicated prior to screening.

The review was restricted to studies published between 2020 and 2025 for two reasons. First, this five-year window reflects contemporary practice and captures recent population-specific validations and method modifications that have emerged following earlier foundational work. Moreover, the performance of the established methods continues to be reported and updated in recent literature, making this period most relevant to current clinical decisions. Second, restricting the window to a defined recent interval reduces heterogeneity in reporting practices and contextual factors (e.g., digital radiography equipment and standards, software tools) compared with searches spanning several decades.

Study selection and eligibility criteria.

Two reviewers (the first and second authors) independently screened titles and abstracts, followed by full-text assessment against predefined eligibility criteria. Disagreements were resolved through discussion; if consensus could not be reached, a third reviewer adjudicated (TM-B).

The inclusion criteria were as follows:

1. Original studies evaluating dental age estimation in children and/or adolescents using panoramic radiographs.
2. Studies including participants within the pediatric/adolescent age span (0–18 years), including mixed cohorts extending into young adulthood, provided that pediatric/adolescent data were included in the analysis.

Exclusion criteria:

1. Sample size below 400 individuals (pragmatic restriction applied to prioritize larger cohorts and more stable descriptive comparisons).
2. Studies based on overlapping populations; when population overlap was suspected, only one study from the same population was retained to avoid duplication.
3. Studies evaluating imaging modalities other than panoramic radiography only.
4. Studies using artificial intelligence algorithms for dental age estimation without reference to a classical dental age estimation method.
5. Studies conducted exclusively in individuals with growth disorders or developmental anomalies.
6. Studies based entirely on a limited number of teeth only (e.g., third molars only).

**Fig. 1:**
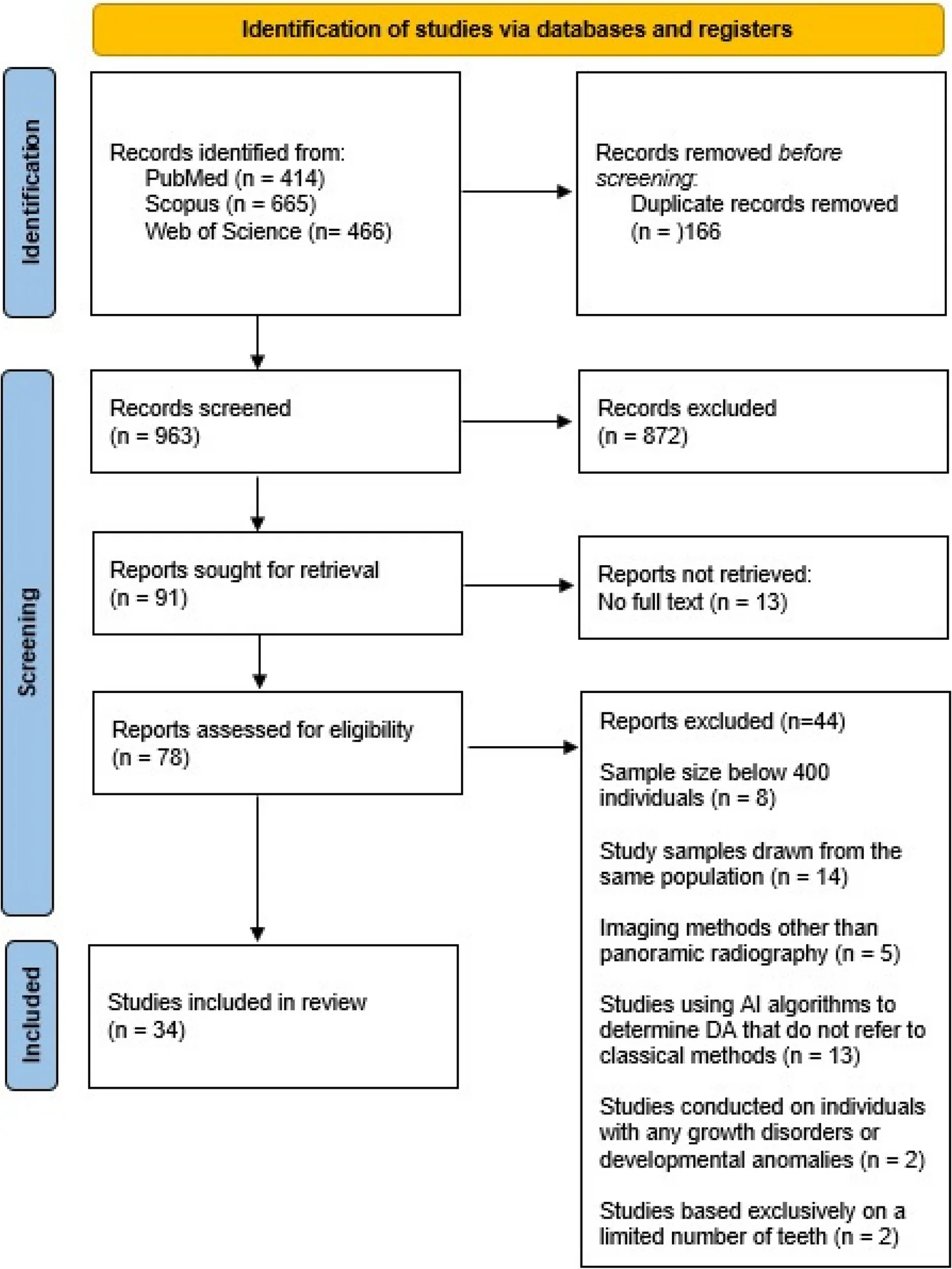
PRISMA flow diagram of the included studies

After removal of 166 duplicates, 963 records were identified. Manual screening of titles and abstracts resulted in 91 articles selected for full-text analysis. Full texts were unavailable for 13 studies. In total, 78 articles were assessed for eligibility, of which 44 were excluded. Ultimately, 34 studies were included in the review (Fig. 1).

Excluded reports are presented in a Supplementary Table S1.

### Screening and data charting

Data charting was performed independently and in duplicate by two reviewers (MŁ-M and WK) using a standardized charting form piloted on a random subset of five included studies before full data charting. The form was refined after pilot testing to ensure consistent capture of study characteristics, methodological details, dental age estimation methods, and accuracy outcomes. Disagreements in charted data were resolved through discussion; where consensus could not be reached, a third reviewer (TM-B) adjudicated.

Extracted variables included first author and year; country/region and population descriptors; study setting/design; sample size (total and sex-stratified where available); age range; dental age estimation method(s); and reported accuracy outcomes. Accuracy measures were recorded as provided by the authors, prioritizing mean absolute error (MAE, years) and mean difference/bias (dental age (DA) minus chronological age (CA), years), and whether results were reported overall or stratified by sex and/or age groups.

For descriptive synthesis of method usage, methods were grouped at the family level (e.g., Willems-family, Demirjian-family, Cameriere-family), while modified or population-specific versions (e.g., Willems I/II, Willems SA/BC, modified Cameriere equations) were retained as variants in the detailed accuracy tables. This approach was adopted to avoid double counting conceptually related methods in frequency analyses.

### Data items

The following data items were charted: first author, publication year, country or region, population characteristics, study design and setting, sample size, sex distribution, chronological age range, sampling strategy, date-of-birth verification, dental age estimation method or method family, method variant where applicable, reported accuracy outcomes, sex-stratified outcomes where available, interrater reliability, intrarater reliability, and authors’ main conclusions. Accuracy outcomes were recorded as reported by the original authors, with priority given to mean absolute error (MAE) and directional bias expressed as dental age minus chronological age (DA–CA). Where original studies reported chronological age minus dental age (CA–DA), values were converted to DA–CA for consistency.

For descriptive synthesis of method usage, methods were grouped at the family level (e.g., Willems-family, Demirjian-family, Cameriere-family), while modified or population-specific versions (e.g., Willems I/II, Willems SA/BC, modified Cameriere equations) were retained as variants in the detailed accuracy tables. This approach was adopted to avoid double counting conceptually related methods in frequency analyses.

### Risk of bias assessment

Two reviewers independently evaluated methodological quality and risk of bias using an adapted Newcastle–Ottawa Scale (NOS) for cross-sectional studies [13]. The adapted scale comprised four evaluated domains: Clarity of stated aim (0–2), Sample selection (0–8), Comparability (0–2), and Outcome (0–4), with a total score 0–16.

Studies were classified according to the total score as follows: 13–16 points indicated high methodological quality and low risk of bias, 9–12 points indicated moderate quality and moderate risk of bias, while scores below 9 points reflected low quality and a high risk of bias. Any discrepancies between reviewers were resolved through discussion and, when necessary, consultation with a third reviewer (TM-B).

## Results

### Characteristics of included studies

This scoping review included 34 studies examining the reported accuracy of panoramic radiograph–based dental age estimation methods across diverse populations (Table 1). Most studies were retrospective and clinic-based, although population-based datasets were also represented. The included studies comprised more than 35,000 children and adolescents from 20 countries/regions, with sample sizes ranging from 400 to 5,017. Age ranges varied substantially across studies, and several cohorts extended into young adulthood while still including pediatric/adolescent participants.

**Table 1:** Baseline characteristics of the included studies

Authors	Country/region	Study design/setting	Sample size	Chronological age range, (mean age ± SD)	Methods evaluated	Sampling type	Date of birth verification
AlOtaibi, N. et al. 2023 [14]	Saudi Arabia (Saudi population)	Retrospective; Population-based	Total: 400 Male: 200 Female: 200	6.00-15.99 years	Moorrees, Fanning, and Hunt (MFH) as adapted by Smith; GH; Nicodemo	Convenience	Official record
AlOtaibi, N. et al. 2023 [15]	Saudi Arabia (Saudi population)	Retrospective; Clinic-based	Total: 400 Male: 200 Female: 200	6-15.99 years	MFH, Demirjian, GH, Nolla, Chaillet, Nicodemo	Consecutive	Official record
Alqerban, A. et al. 2021 [16]	Saudi Arabia (Saudi Arabian)	Retrospective; Clinic-based	Total: 1146 Male: 605 Female: 541	4–18 years	Willems BC, Willems SA	Convenience	Official record
Bakhsh, H. et al. 2024 [17]	Saudi Arabia (Saudi population)	Retrospective; Clinic-based	Total: 711 Male: 357 Female: 354	5–15 years	Demirjian, London Atlas	Random	Official record
Bedek, I. et al. 2022 [18]	Croatia (Croatian population)	Retrospective; Population-based	Total: 1996 Male: 1121 Female: 875	5–16 years	Demirjian, Willems, Haavikko	Convenience	NR
Briem Stamm, A. et al. 2022 [19]	Argentina (Autonomous City of Buenos Aires)	Retrospective; Clinic-based	Total: 508 Male: 240 Female: 268	6–14 years	Demirjian	Convenience	Official record
Brkic, H. al. 2022 [20]	Croatia (Zagreb metropolitan area)	Retrospective; Population-based	Total: 1492 Male: 704 Female: 788	6.0-13.9 years	Cameriere, Demirjian, Haavikko, Willems	Consecutive	Self-reported
Dalben, E. et al. 2022 [21]	Brazil (Central-West Brazil)	Retrospective; population-based	Total: 1,104 Male: 595 Female: 509	6-15.99 years (10.88 ± 2.84 years)	London Atlas	Convenience	NR.
Darıcı, A. et al. 2024 [22]	Finland, Turkey (Finnish, Turkish)	Retrospective; Population-based	Total: 905 Male: 443 Female: 452	5–15 years	Cameriere, Demirjian	Random	Official record
Duruk, G. al. 2022 [23]	Turkey (Eastern Turkish population)	Retrospective; Clinic-based	Total: 1587 Male: 813 Female: 774	3-17.9 years	Demirjian, Nolla	Convenience	Official record
El-Desouky, S. al. 2023 [24]	Egypt (Egyptian)	Prospective; population-based	Total: 762 Male: 408 Female: 354	5–15 years	Cameriere, Willems	Convenience	Official record
Franco, A. et al. 2024 [25]	Brazil (mixed ancestry: Native Indians, Africans, Western Europeans)	Retrospective; Clinic-based	Total: 5017 Male: 2443 Female: 2574	6-15.99 years (10.99 ± 2.76 years)	Willems, Demirjian	Convenience	Official record
Gonçalves do Nascimento, L. et al. 2020 [26]	Brazil (north east Brazilians)	Retrospective; Clinic-based	Total: 429 Male: 188 Female: 241	5-14.99 years (12.02 ± 2.06)	Cameriere	Random	Official record
Gümüşboğa Z. et al. 2024 [27]	Turkey (eastern Turkey)	Retrospective; Clinic-based	Total: 1018 Male: 497 Female: 521	5–14 years	Bedek models, Cameriere-European Formula (CF)	Convenience	Official record
Gupta, S. et al. 2023 [28]	India (North Indian population, specifically Uttar Pradesh)	Retrospective; Population-based	Total: 762 Male: 329 Female: 433	7–16 years	Cameriere, Demirjian	Random	Official record
Hato, E. et al. 2022 [29]	Turkey (Turkish children in the Central Black Sea Region)	Retrospective; Clinic-based	Total: 400 Male: 196 Female: 204	6–14 years	Nolla, Willems, Cameriere	Convenience	Official record
Ishwarkumar, S. et al. 2022 [30]	South Africa (KwaZulu-Natal, South African Black and Indian population groups)	Retrospective; population-based	Total: 840; South African Black: 420; South African Indian: 420;	5.00-15.99 years	Cameriere	Consecutive	Official record
Kar, M. et al. 2025 [31]	India (West Bengal) (Bengali)	Retrospective; Population-based	Total: 411, Male: 204, Female: 207	6–14 years (9.9 ± 2.19 years)	Willems, Cameriere	Convenience	NR
Koç, A et al. 2021 [32]	Turkey (Eastern Turkish, various ethnic backgrounds)	Retrospective; Clinic-based	Total: 919 Male: 460 Female: 459	6–14 years (9.88 ± 2.17 years)	Willems, London Atlas, Nolla	Convenience	Official record
Kuppusamy R. et al. 2025 [33]	Malaysia (multiethnic population)	Retrospective; Clinic-based	Total: 523 Male: 279 Female: 244	4-16.99 years old	London Atlas	Convenience	NR
Kuremoto, K. et al. 2022 [34]	Japan (Japanese nationals)	Retrospective; Clinic-based	Total: 1024, Male: 512, Female: 512	3–18 years	Haavikko’s	Convenience	Official record
Kwon, K. et al. 2023 [35]	China (Han population in Shanghai)	Retrospective; Clinic-based	Total: 634 Male: 319 Female: 311	8.00-14.99 years	Demirjian, Willems	Random	NR
Lin, Y. et al. 2022 [36]	China (Chinese Uyghur population)	Cross-sectional; Clinic-based	Total: 831 Male: 354 Female: 477	10.00-16.99 years	London Atlas, Willems, New Quick Method	Random	NR
Moca, A. et al. 2022 [37]	Romania (Oradea)	Retrospective; Clinic-based	Total: 1006 Male: 431 Female: 575	3-13.9 years (9.496 ± 2.218 years)	Demirjian	Convenience	Official record
Moga, L. et al. 2025 [38]	Romania (northwestern Romania)	Retrospective; Clinic-based	Total: 860 Male: 356 Female: 504	3-15.9 years (mean: 9.95 years)	Nolla	Convenience	Official record
Pan, J et al. 2021 [39]	China (eastern China)	Retrospective; clinic-based	Total: 2367, Male: 1217, Female: 1150	5–16 years (boys 11.20 ± 3.29 years; girls 10.99 ± 3.12 years)	Demirjian, Willems, Modified Demirjian	Convenience	Official record
Şahin, T. et al. 2025 [40]	Turkey (Central Anatolia region, diverse ethnic backgrounds)	Retrospective; Clinic-based	Total: 616 Male: 295 Female: 321	6-14.99 years	Cameriere, Modified Cameriere, Willems, Blenkin-Evans	Convenience	Official record
Sgheiza, V. et al. 2023 [41]	United Kingdom (London, Bangladeshi and European ancestry)	Retrospective; Population-based	Total: 3334 Male: NR Female: NR	2–23 years	Moorreeset al. Demirjian	Systematic sampling (stratified by age, sex, and ancestry)	NR
Shan, W. et al. 2022 [42]	South China (population)	Retrospective; Clinic-based	Total: 1,477 Male: 644 Female: 833	2.00-17.99 years	Willems, Demirjian	Convenience	Official record
Sheriff, S. O. et al. 2020 [43]	South India (South Indian population)	Retrospective; Clinic-based	Total: 650 Male: 275 Female: 375	7–15 years	Bedek, Willems	Convenience	NR
Valente, R. et al. 2024 [44]	Brazil (central-western region)	Retrospective; population-based	Total: 3,271 Male: 1,637 Female: 1,634	6-22.9 years (mean 14.6 ± 4.9 years)	Nicodemo (NMM)	Convenience	Official record
Wang, J. et al. 2022 [45]	China (Eastern Chinese, Chinese Han population)	Retrospective; Clinic-based	Total: 1211 Male: 582 Female: 629	11–16 years old	Willems I, Willems II	Random from a Convenience sample	NR
Yavuz, B. et al. 2025 [46]	Turkey (Turkish population, northwestern Turkey)	Prospective; Clinic-based	Total: 1169 Male: 556 Female: 613	5–16 years	Willems, Cameriere-Europe, London Atlas, Deep Learning	Convenience	Official record
Zhou J. et al. 2023 [47]	China (East China)	Retrospective; Clinic-based	Total: 835, Male: 400, Female: 435	6.0-19.9 years	London Atlas, Demirjian	Convenience	NR

*NR *not reported

### Risk of bias assessment

The risk of bias assessment using the adapted Newcastle–Ottawa Scale is summarized in Table 2, with detailed data presented in Supplementary Table S2. Across the 34 included studies, NOS scores ranged from 9 to 13 out of 16, with a median score of 12 and a mean score of 11.6. No study was classified as low quality or high risk of bias. Seven studies were rated as high methodological quality and low risk of bias, whereas 27 studies were rated as moderate methodological quality and moderate risk of bias (Table 2). Risk-of-bias ratings were not used to exclude studies from the review; they were used to contextualize the descriptive synthesis.

**Table 2:** Summary of risk of bias assessment

NOS category	NOS score range	Number of studies	Interpretation
High quality / low risk of bias	13–16	7/34, 20.6%	Low risk of bias
Moderate quality / moderate risk of bias	9–12	27/34, 79.4%	Moderate risk of bias
Low quality / high risk of bias	< 9	0/34, 0%	High risk of bias

Overall, the included studies showed generally acceptable methodological quality for descriptive synthesis. The strongest domains were clarity of the stated aim, exposure assessment, handling of non-respondents, and assessment of the outcome, which were consistently scored highly across the included studies. The main recurrent methodological limitations concerned sample representativeness and comparability. Most studies were based on retrospective, clinic-based, or convenience samples, which limits the generalizability of their findings to broader pediatric and adolescent populations.

Because most included studies evaluated two or more method families simultaneously, risk-of-bias ratings were assigned at the study level rather than at the method level. Nonetheless, to facilitate comparison across the four most prevalent method families, the distribution of NOS quality categories was examined within the subset of studies that evaluated each method. Among studies reporting Willems-family methods (*n* = 15), five were rated high quality and ten moderate quality, corresponding to a mean NOS score of 11.9. Among studies reporting Demirjian-family methods (*n* = 13), two were rated high quality and eleven moderate quality (mean NOS 11.4). Among studies reporting Cameriere-family methods (*n* = 11), four were rated high quality and seven moderate quality (mean NOS 12.1). Among studies reporting London Atlas methods (*n* = 7), three were rated high quality and four moderate quality (mean NOS 12.0). The NOS score distributions were broadly similar across all four method families, indicating that risk-of-bias considerations do not systematically favour one method over another in the descriptive accuracy comparisons presented below. The main driver of lower scores across all groups was the use of clinic-based or convenience sampling rather than population-representative sampling frames.

### Frequency of dental age assessment methods

**Fig. 2:**
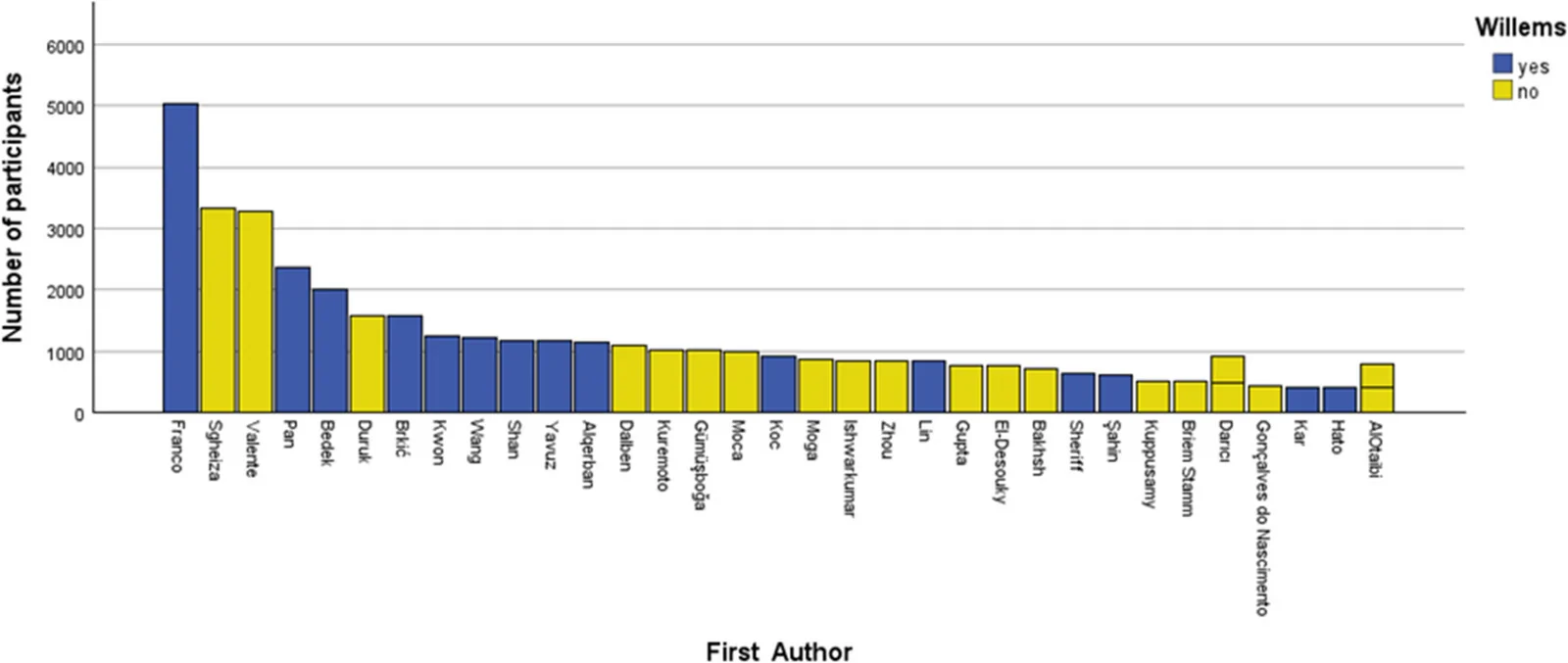
Summary of the 12 authors who analyzed the largest patient cohorts, with identification of studies employing the Willems method

When method usage was analyzed descriptively at the family level, Willems-family methods were the most frequently reported, followed by Demirjian-, Cameriere-, and London Atlas-based approaches (Table 3; Fig. 2). This pattern was consistent when considering both the number of studies and the cumulative number of patients, although the exact ranking varied slightly depending on whether studies, countries, or patient totals were used as the denominator.

**Table 3:** Distribution of dental age estimation methods at the family level across studies, countries, and patients

Method of Dental Age Estimation	Study Prevalence (Valid Percent [%])	Country Prevalence (Valid Percent [%])	Patient Prevalence (Valid Percent [%])
Willems	15 (23,4)	7 (15,9)	20,745 (30,0)
Demirjian	12 (18,8)	7 (15,9)	13,772 (19,9)
Cameriere	11 (17,2)	8 (18,2)	8465 (12,2)
London Atlas	7 (10,9)	6 (13,6)	6092 (8,8)
Nolla	5 (7,8)	3 (6,8)	4166 (6,0)
Haavikko	3 (4,7)	2 (4,5)	4596 (6,6)
Moorrees	3 (4,7)	3 (6,8)	4134 (6,0)
Nicodemo	2 (3,1)	2 (4,5)	3671 (5,3)
GH	2 (3,1)	1 (2,3)	800 (1,2)
Bedek	2 (3,1)	2 (4,5)	1668 (2,4)
Chaillet	1 (1,6)	1 (2,3)	400 (0,6)
Blenkin-Evans	1 (1,6)	2 (4,5)	616 (0,9)
Total	64 (100)	44 (100)	69,125 (100)

### Diagnostic accuracy by method

Across the included studies, reporting of accuracy measures was heterogeneous. Out of 75 study-method comparisons summarized in Table 4, sex-stratified MAE was reported in 33/75 (males) and 33/75 (females), whereas sex-stratified mean difference (DA-CA) was available in 64/75 (males) and 64/75 (females). The summary of diagnostic accuracy metrics provided in all of the analyzed studies is presented in Table 4.

**Table 4:** Detailed accuracy measures

First Author	Method of Age Estimation	Mean Absolute Error (MAE)			Estimated age difference (DA – CA)			Interrater Reliability (ICC, kappa value) ^a^	Intrarater Reliability (ICC, kappa value)^a^	Conclusion
		Male [years]	Female [years]	Total sample [years]	Male [years]	Female [years]	Total sample [years]			
AlOtaibi, N. N et al. 2023 [14]	MHF adapted by Smith	NR	NR	NR	-0.82	-0.7	-0.65	NR	Kappa = 0.88	Nicodemo et al.‘s method had the lowest bias for all teeth except for the lower first molar. GH had the lowest bias for the lower first molar. MFH’s staging system was more representative of dental development for an individual tooth. Most accurate teeth for MFH: lower central and lateral incisors. Most accurate teeth for Nicodemo et al.: upper central incisor and lower first premolar. Least accurate tooth: lower first molar.
	GH	NR	NR	NR	NR	NR	NR		Kappa = 1	
	Nicodemo	NR	NR	NR	-0.06	-1.03	-0.545*		Kappa = 0.97	
Sheriff, S. O et al. 2020 [43] **	Bedek	0.85	0.88	0.86	NR	NR	NR	NR	NR	Bedek et al’s new models performed better than the Willems models in terms of MAE and percentage of correct estimations. The 7 tooth and 4 tooth models showed high accuracy, with the 6 tooth model overestimating in boys. These models are suitable for South Indian children, particularly in cases of hypodontia or missing teeth.
	Willems	1,02	1.25	1.16	NR	NR	NR			
Pan, J et al. 2021 [39]	Demirjian	0.73	0.79	0.7592*	+ 0.21	+ 0.1	+ 0.157*	0.728	0.756	The modified method was more accurate than the Demirjian and Willems methods for eastern Chinese children. The Willems method was more accurate than the Demirjian method but less accurate than the modified method.
	Willems	0.7	0.79	0.744*	-0.07	-0.24	−0.153*			
	Modified Demirjian	0.63	0.64	0.635*	+ 0.04	+ 0.09	+ 0.064*			
Alqerban, A et al. 2021 [16]	Willems SA	1.37	1.37	1.37	+ 0.023	+ 0.06	0.0373*	0.919	0.983	The Willems SA method and Willems BC method have very similar performance in estimating dental age in Saudi children, with minimal differences in error. Belgian Caucasian reference data can be used effectively in the Saudi population, indicating no need for a Saudi Arabian-specific model
	Willems BC	1.35	1.32	1.33	-0.01	+ 0.06	0.02			
Duruk, G et al. 2022 [23]	Demirjian	NR	NR	NR	+ 0.61	+ 0.75	+ 0.68	kappa = 0.95	Kappa = 0.98-1.0	Nolla’s method was more accurate than Demirjian’s method for estimating chronological age in the Eastern Turkish population. It underestimated chronological age, while Demirjian’s method overestimated it. The authors suggest using Nolla’s method primarily for estimating chronological age in Turkish children
	Nolla	NR	NR	NR	-0.003	-0.32	-0.16	kappa = 0.94	Kappa = 0.97–0.98	
Bedek, I et al. 2022 [18]	Demirjian	NR	NR	NR	+ 0.8	+ 0.84	+ 0.8195*	NR	Kappa = 0.92	The Willems method is the most accurate for dental age estimation in Croatian children, with the Demirjian and Haavikko methods showing greater deviations and requiring adaptation for this population.
	Willems	NR	NR	NR	+ 0.41	+ 0.22	+ 0.3173*		NR	
	Haavikko	NR	NR	NR	-0.61	-0.82	-0.7124*		Kappa = 0.83	
Dalben, E et al. 2022 [21]	London Atlas	1.19	1.22	1.21	+ 0.6	+ 0.56	+ 0.58	0.98	0.98 and 0.99	The London Atlas Software App 2nd edition performed well for dental age estimation in Brazilian children, with mean error (bias) errors of 0.56 and 0.60 years for females and males, respectively. The method is acceptable for clinical applications, especially in the age range of 6 to 9.99 years. However, caution is advised in forensic applications during late childhood to early adolescence due to the role of third molars.
Shan, W et al. 2022 [42]	Demirjian	0.96	0.95	0.955	+ 0.42	+ 0.31	+ 0.4492*	0.963	0.930	The Willems method was more accurate than the Demirjian method for the southern China population. The GBDT algorithm significantly improved the accuracy of dental age estimation compared to traditional methods.
	Willems	0.77	0.78	0.775	+ 0.09	-0.16	-0.064*			
Hato, E et al. 2022 [29]	Nolla	NR	NR	NR	-0.24	-0.27	-0.2553*	ICC = 0.95	ICC = 0.995	The Cameriere method provided the closest estimation to chronological age for both girls (79.9%) and boys (80.6%) in the study population, followed by the Nolla method. Both Cameriere and Nolla methods can be used for Turkish children in the Central Black Sea Region.
	Willems	NR	NR	NR	+ 0.49	+ 0.76	+ 0.6277*	ICC = 0.885	ICC = 0.997	
	Cameriere	NR	NR	NR	-0.17	-0.1	-0.135*	ICC = 0.978	ICC = 0.999	
Lin, Y et al. 2022 [36]	London Atlas	0.83	0.88	0.86	-0.06	-0.05	-0.06	ICC = 0.928	ICC = 0.942	The London Atlas outperformed the Willems method with better accuracy and precision among 10–15 years Chinese Uyghur children. The new quick method is comparable to the London Atlas for children aged 10–14 and is more time-saving and convenient.
	Willems	1.16	1.17	1.165	-0.82	-0.85	-0.44	ICC = 0.91	ICC = 0.885	
	Quick method	0.71	0.69	0.70	-0.16,	-0.09	-0.30	ICC = 0.901	ICC = 0.922	
Ishwarkumar, S et al. 2022 [30]	Cameriere	1.02	1.03	0.1025*	+ 0.47	+ 0.585	NR	0.97	0.99	The population-specific regression formulas developed using stepwise regression analysis and a best-fit model performed best for the South African Black and Indian population groups, showing excellent correlation between chronological and dental ages with R² values greater than 0.9.
	Bayesian SA Black Cameriere	0.84	0.84	0.84*	-0.17	-0.21	NR			
Brkic, H et al. 2022 [20]	Cameriere	0.50	0.51	0.505	-0.05	-0.04	-0.4	ICC = 0.89	ICC = 0.91	The Cameriere European formula performed best for estimating dental age in Croatian children, showing the highest accuracy and precision compared to chronological age. It is recommended as the first choice for forensic and clinical analyses. The Haavikko method is also recommended due to its good accuracy and precision.
	Haavikko	0.61	0.61	0.61	-0.04	-0.3	-0.17	ICC = 0.78	ICC = 0.77	
	Demirjian	1.01	1.18	1.095	+ 0.94	+ 1.09	+ 1.02	ICC = 0.78	ICC = 0.81	
	Willems	0.78	0.7	0.74	+ 0.61	+ 0.37	+ 0.48	NR	NR`	
Briem Stamm, A. D et al. 2022 [19]	Demirjian	NR	NR	NR	NR	NR	NR	NR	NR	The Demirjian method overestimated dental age compared to real age in the study population from Buenos Aires City, indicating a need for complementary indicators of biological maturity due to individual variations in dental maturity.
Moca, A. E et al. 2022 [37]	Demirjian	NR	NR	NR	+ 1.46	+ 1.417	1.4354*	NR	ICC = 0.996	The Demirjian method overestimated the age of Romanian children and requires adaptations for accurate use in this population.
Moga, L. I et al. 2025 [38]	Nolla	NR	NR	NR	-1.256	-1.707	-1.52	NR	NR	The Nolla method consistently underestimated dental age in Romanian children, with greater discrepancies in older age groups and among girls. It requires population-specific calibration to improve accuracy.
Şahin, T. N et al. 2025 [40]	Cameriere	NR	NR	NR	+ 0.17	+ 0.22	0.196*	ICC above 0.9	ICC above 0.9	The Cameriere method (CM) provided the closest results to chronological age (CA) when evaluating groups separately. The Blenkin-Evans method (BEM) gave the closest results to CA when considering all age groups together by gender. CM and BEM showed higher accuracy rates compared to MCM and WM. MCM requires further validation for the Turkish population.
	Modified Cameriere	NR	NR	NR	-0.52	-0.49	-0.5044*			
	Willems	NR	NR	NR	-0.43	-0.45	-0.4404*			
	Blenkin-Evans	NR	NR	NR	-0.12	-0.20	-0.1607*			
El-Desouky, S. S et al. 2023 [24]	Cameriere	NR	NR	NR	-0.53	-0.59	-0.5579*	NR	Kappa above 0.85	The Egyptian-specific formula was more accurate than Cameriere’s original formula for dental age estimation in Egyptian children, with better performance in boys than girls.
	New Egyptian formula	NR	NR	NR	-0.12	+ 0.1	-0.0178*			
Franco, A et al. 2024 [25]	Willems	0.79	0.81	0.80	NR	NR	NR	Weighted Kappa 0.83	Weighted Kappa 0.91	The Brazilian model (FRANCO) performed better than Willems’ method for the study population, with lower mean absolute errors (MAE) and root mean squared errors (RMSE), and reduced MAE in 70% of the age categories.
	FRANCO model	0.72	0.74	0.73	NR	NR	NR			
Gonçalves do Nascimento et al. 2020 [26]	Cameriere	NR	NR	NR	-0.32	-0.3	-0.31	ICC 0.891	ICC 0.975	The method generally underestimated chronological age by approximately 0.3 years for both males and females. Overestimation occurred in younger groups (5-6.99 and 7-8.99 years), while underestimation was significant in the 13-14.99 years group. A correction factor improved predictive power to approximately 80%. The method is applicable for ages 5 to 13 years, with reduced applicability near the age of 15. For females, use the general equation; for males, use the male-specific equation. Combine methods for the 13-14.99 years age group for better accuracy.
Zhou, J et al. 2023 [47]	London Atlas	0.58	0.58	0.58	NR	NR	NR	NR	NR	The London Atlas method performed better than the Demirjian method in terms of reproducibility, repeatability, and agreement with chronological age in the East China population. It is more universally applicable but may not fully meet all needs for age estimation.
	Demirjian	0,66	0,75	0.7069*	NR	NR	NR			
Gupta, S et al. 2023 [28]	Cameriere	NR	NR	NR	-0.14	-0.28	-0.2*	0.897	0.961	Both modified Demirjian and Cameriere methods performed better than their original counterparts for the north Indian population, with the modified versions showing higher accuracy and better clinical applicability.
	Demirjian	NR	NR	NR	1.21	+ 1.72	+ 1.4289*	0.975	0.951	
Kwon, K et al. 2023 [35] ***	Demirjian	0.7	0.77	0.7346*	-0.51	-0.48	-0.4952*	0.753	0.729	The Demirjian method was more accurate than the Willems method for the study population. The Demirjian method overestimated dental age in 2009–2011 and underestimated it in 2021, while the Willems method overestimated for males and underestimated for females in 2009–2011, and underestimated for both genders in 2021
	Willems	0.88	0.99	0.9343*	-0.8	-0.82	-0.8099*			
AlOtaibi, N. N et al. 2023 [15]	Chaillet	NR	NR	NR	-2.03	-2.35	-2.19	0.81	0.96	Demirjian’s method was the most accurate. MFH method was the second most accurate. Nicodemo et al. and Chaillet et al. methods were the least accurate methods.
	Demirjian	NR	NR	NR	+ 0.18	+ 0.11	+ 0.15	0.81	0.96	
	GH	NR	NR	NR	-0.84	-1.16	-1.0	0.87	1.0	
	MHF	NR	NR	NR	-0.88	-1.14	-1.01	0.80	0.88	
	Nolla	NR	NR	NR	-1.07	-1.5	-1.29	0.78	0.97	
	Nicodemo	NR	NR	NR	-1.8	-1.65	-1.72	0.73	0.97	
Bakhsh, H. H et al. 2024 [17]	Demirjian	NR	NR	NR	+ 0.55	+ 0.76	+ 0.6546*	81% Cohen’s kappa	NR	The London Atlas (LAE) method is more accurate than the Demirjian (DE) method for estimating dental age in a Saudi population of children and adolescents aged 5–15 years. LAE showed minimal mean differences from chronological age and no statistically significant differences, while DE consistently overestimated dental age.
	London Atlas	NR	NR	NR	+ 0.03	-0.002	+ 0.014*			
Gümüşboğa Z. S et al. 2024 [27] **	Bedek	0.7	0.7	0.7*	0	+ 0.1	+ 0.04	ICC = 0.87	ICC = 0.97	The Cameriere-European Formula (CF) method is more successful in age estimation compared to the Bedek models for children in eastern Turkey. Among the Bedek models, the three-tooth model is the closest predictor of tooth age in the whole sample. The four-tooth model is most appropriate for boys, and the two-tooth model is most appropriate for girls within the Bedek models. The CF method is particularly accurate for the age range of 13-13.9 years.
	Cameriere	0.6	0.6	0.6*	-0.2	+ 0.3	+ 0.1	NR	NR	
Darıcı, A et al. 2024 [22]	Cameriere Finnish	NR	NR	NR	-0.352	-0.479	-0.4115*	ICC = 0.65	ICC = 0.57	Demirjian’s method generally overestimated dental age across cohorts except in 8-year-old girls, whereas Cameriere’s method predominantly underestimated dental age, with the exception of 6-year-old girls and boys. Among Northern Finnish children, Demirjian’s method yielded more accurate estimates for boys, while Cameriere’s method performed better for girls. Comparative analysis indicated higher dental ages in Turkish than Finnish children, with significant discrepancies between dental and chronological ages in both populations overall and particularly pronounced in 10-year-old boys and 8-year-old girls using both methods.
	Cameriere Turkish	NR	NR	NR	-0.237	-0.235	-0.2359*	0.99	ICC = 0.99	
	Demirjian Finnish	NR	NR	NR	+ 0.469	+ 0.216	+ 0.3504*	Kappa = 0.41	Kappa = 0.79	
	Demirjian Turkish	NR	NR	NR	+ 0.638	+ 0.552	+ 0.5921*	Kappa = 0.84	Kappa = 0.96	
Kar, M et al. 2025 [31]	Willems	NR	NR	NR	NR	NR	+ 0.286	Kappa = 0.8	NR	Willems method overestimated dental age by 0.286 years. Cameriere method showed no significant difference between software tools. Both methods are equally applicable and reliable for age estimation in the study population.
	Cameriere CD	NR	NR	NR	NR	NR	-0.139			
	Cameriere SD	NR	NR	NR	NR	NR	-0.1			
Koç, A et al. 2021 [32]	London Atlas	0.79	0.87	0.83	-0.073	+ 0.284	+ 0.11	ICC ≥ 0.948	ICC ≥ 0.948	The Willems method is the most accurate for estimating dental age in Eastern Turkish children, with a mean prediction error of 0.80 years. The London Atlas method is the second most accurate, with a mean prediction error of 0.83 years, and is noted for its practicability and comparable accuracy. The Nolla method is the least accurate.
	Willems	0.79	0.81	0.8	+ 0.4286 + 0.29	+ 0.339 + 0.34	+ 0.31			
	Nolla	0.84	0.94	0.89	-0.411	-0.567	-0.49			
Kuppusamy, R et al. 2025 [33]	London Atlas	0.5821	0.603	0.5937	-0.002	-0.08	-0.0365	Cohen’s Kappa = 0.693	ICC2.1: 0.992, ICC absolute:0.976 - Cohen’s Kappa: 0.769	The London Atlas of Human Tooth Development and Eruption showed high accuracy in Malaysian children, with a mean absolute difference of 0.60 years and no significant difference between chronological and estimated ages. It provides consistent estimation for both male and female subjects, with approximately 87% of estimates within one year of chronological age. The method has a low margin of error and is reliable for this population.
Valente, R. P. A et al. 2024 [44]	Nicodemo	NR	NR	NR	-5.1	-5.1	-5.1*	Weighted kappa values above 0.8	Weighted kappa values above 0.8	The Nicodemo’s method is not suitable for individuals older than 12 years due to its poor performance in estimating ages accurately beyond this age. Newer methods like the London Atlas and Cameriere’s are suggested as superior alternatives for adolescents and young adults.
Wang, J et al. 2022 [45]	Willems I	0,95	1	0.975	-0.44	-0.09	NR	NR	NR	Willems I is more accurate for boys. Willems II is more accurate for girls. Neither method is perfectly reliable for the entire age range of 11 to 16 years, particularly for those aged 16 and above.
	Willems II	1.02	1.0	1.01	-0.57	+ 0.09	NR	NR	NR	
Yavuz, B. S et al. 2025 [46]	Willems	0.9006*	1.0098*	0.9579*	0.68	0.68	+ 0.68	0.85	0.95–0.98	The London Atlas is only suitable for boys in predicting the age of Turkish children, Willems, Cameriere-Europe formulas need revision before application in Turkish population.
	Cameriere	0.7674*	0.8058*	0.7875*	-0.31	-0.39	-0.35	0.87	0.94–0.95	
	London Atlas	0.7231*	0.7408*	0.7324*	0.05	0.26	+ 0.16	0.84	0.89 − 0.90	

All accuracy values are reported in years. Positive DA–CA values indicate overestimation, and negative DA–CA values indicate underestimation. Modified or population-specific versions of methods are identified at the variant level where reported by the original authors

*Abbreviations: MAE *mean absolute error, *DA–CA *dental age minus chronological age, *ICC *intraclass correlation coefficient, *NR *not reported

*- calculated on available data; ** -accuracy provided for Bedek’s 7 tooth models; ***- data presented for population from 2021

Across the four most frequently used approaches (Willems, Demirjian, Cameriere, London Atlas), both the magnitude and direction of bias varied by population and method variant, showing that performance is population-dependent and that the “best” method cannot be asserted universally from descriptive summaries alone. To support method-specific interpretation, the results for the four most frequently reported methods are summarized in Tables 4, 5, 6, 7 and 8.

### Demirjian method

The Demirjian method was reported in 13 study–method comparisons (Table 5). Where MAE was reported, sex-stratified MAE ranged from 0.66–1.01 years in males and 0.75–1.18 years in females (Pan; Shan; Brkić; Zhou; Kwon). Bias (DA–CA) was predominantly positive (overestimation), with multiple studies reporting mean overestimation typically on the order of ~+0.55 to +1.46 years depending on population (e.g., Croatia, Romania, Argentina, India, Turkey, Saudi Arabia). Notable exceptions were observed in some Chinese cohorts, where Demirjian underestimated age (e.g. Kwon: −0.51 in boys with +0.48 in girls). Collectively, these findings indicate that Demirjian-derived bias is not stable across populations and can change in both direction and magnitude.

**Table 5:** Studies utilizing the Demirjian method for dental age assessment

Reference	Country/region	Sample size	MAE (M)	MAE (F)	MD DA-CA (M)	MD DA-CA (F)	Direction (M/F)
AlOtaibi, N. N. et al. 2023 [15]	Saudi Arabia (Saudi children)	Total: 400 Male: 200 Female: 200	NR	NR	+ 0.18	+ 0.11	Over/Over
Bakhsh, H. H. et al. 2024 [17]	Saudi Arabia (Saudi population)	Total: 711, Male: 357, Female: 354	NR	NR	+ 0.55	+ 0.76	Over/Over
Bedek, I. et al. 2022 [18]	Croatia (Croatian population)	Total: 1996 Male: 1121 Female: 875	NR	NR	+ 0.80	+ 0.84	Over/Over
Briem Stamm, A. D. et al. 2022 [19]	Argentina (Autonomous City of Buenos Aires)	Total: 508 Male: 240 Female: 268	NR	NR	+ 1.04	+ 1.15	Over/Over
Brkic, H. et al. 2022 [20]	Croatia (Zagreb metropolitan area)	Total: 1492 Male: 704 Female: 788	1.01	1.18	+ 1.02 a	+ 1.02 a	Over/Over
Darıcı, A. et al. 2024 [22]	Turkey (Turkish)	Total: 905 Male: 443 Female: 452	NR	NR	+ 0.64	+ 0.55	Over/Over
	Finland (Finish)		NR	NR	+ 0.469	+ 0.216	Over/Over
Duruk, G. et al. 2022 [23]	Turkey (Eastern Turkish population)	Total: 1587 Male: 813 Female: 774	NR	NR	+ 0.61	+ 0.75	Over/Over
Gupta, S. et al. 2023 [28]	India (North Indian population, specifically Uttar Pradesh)	Total: 762 Male: 329 Female: 433	NR	NR	+ 1.21	+ 1.72	Over/Over
Kwon, K. et al. 2023 [35]	China (Han population in Shanghai)	Total: 634 Male: 319 Female: 315	0.70	0.77	-0.51	-0.48	Under/Under
Moca, A. E. et al. 2022 [37]	Romania (Oradea)	Total: 1006 Male: 431 Female: 575	NR	NR	+ 1.46	+ 1.42	Over/Over
Pan, J. et al. 2021 [39]	China (eastern China)	Total: 2367, Male: 1217, Female: 1150	0.73	0.79	+ 0.21	+ 0.1	Over/Over
Shan, W. et al. 2022 [42]	South China (population)	Total: 1,477 Male: 644 Female: 833	0.95	0.96	+ 0.42	+ 0.31	Over/Over
Zhou, J. et al. 2023 [47]	China (East China)	Total: 835, Male: 400, Female: 435	0.66	0.75	NR	NR	

*NR *not reported

### Cameriere method

The Cameriere method (including modified and/or population-specific equations) was reported in 14 study–method comparisons (Table 6). Where the MAE was available, the reported sex-stratified MAE ranged from 0.50–1.02 years in males and 0.51–1.03 years in females (e.g., Brkić; Gümüşboğa; Ishwarkumar). Across cohorts, bias (DA–CA) tended to be closer to zero than in Demirjian in several datasets but remained method- and population dependent, spanning underestimation (e.g., et al. Darıcı: ~−0.24/−0.23; Hato: ~−0.17/−0.10; Yavuz: ~−0.31/−0.39) and overestimation in some variants/populations (e.g., et al. Ishwarkumar population specific: +0.47/+0.58; Şahin: +0.17/+0.22). Importantly, some papers reported multiple Cameriere-based equations with materially different bias (e.g. et al. Egyptian formula vs. original; modified vs. original Cameriere), so interpretation should be tied to the specific equation used rather than treating “Cameriere” as a single fixed model.

**Table 6:** Studies utilizing the Cameriere method (including modified/population-specific formulas) for dental age assessment

Reference	Country/region	Sample size	MAE (M)	MAE (F)	MD DA-CA (M)	MD DA-CA (F)	Direction (M/F)
Brkic, H. et al. 2022 [20]	Croatia (Zagreb metropolitan area)	Total: 1492 Male: 704 Female: 788	0.50	0.51	-0.4 a	-0.4 a	Under/Under
Darıcı, A. et al. 2024 [22]	Turkey (Turkish)	Total: 905 Male: 443 Female: 452	NR	NR	-0.24	-0.23	Under/Under
	Finland (Finnish)		NR	NR	−0.352	−0.479	Under/Under
El-Desouky, S. S. et al. 2023 [24]	Egypt (Egyptian)	Total: 762 Male: 354 Female: 408	NR	NR	-0.53	-0.59	Under/Under
Gonçalves do Nascimento, L. et al. 2020 [26]	Brazil (north east Brazilians)	Total: 429/Male: 188/Female: 241	NR	NR	-0.32	-0.30	Under/Under
Gupta, S. et al. 2023 [28]	India (North Indian population, specifically Uttar Pradesh)	Total: 762 Male: 329 Female: 433	NR	NR	-0.14	-0.28	Under/Under
Gümüşboğa, Z. S. et al. 2024 [27]	Turkey (eastern Turkey)	Total: 1018 Male: 497 Female: 521	0.60	0.60	-0.20	+ 0.30	Under/Over
Hato, E. et al. 2022 [29]	Turkey (Turkish children in the Central Black Sea Region)	Total: 400 Male: 196 Female: 204	NR	NR	-0.17	-0.10	Under/Under
Ishwarkumar, S. et al. 2022 [30]	South Africa (KwaZulu-Natal, South African Black and Indian population groups)	Total: 840; South African Black: 420; South African Indian: 420;	1.02	1.03	+ 0.47	+ 0.58	Over/Over
Kar, M. et al. 2025 [31]	India (West Bengal) (Bengali)	Total: 411, Male: 204, Female: 207	NR	NR	NR	NR	
Yavuz, B. S. et al. 2025 [46]	Turkey (Turkish children, northwestern Turkey)	Total: 1169 Male: 556 Female: 613	0.81*	0.77*	-0.31	-0.39	Under/Under
Şahin, T. N. et al. 2025 [40]	Turkey (Central Anatolia region, diverse ethnic backgrounds)	Total: 616 Male: 295 Female: 321	NR	NR	+ 0.17	+ 0.22	Over/Over

*NR *not reported

### Willems method

The Willems method was reported most frequently, with 17 study–method comparisons (Table 7). Sex-stratified MAEs were available in a subset and ranged from 0.70–1.37 years in males and 0.70–1.37 years in females, with higher errors typically reported in specific populations or variants (e.g., Saudi Willems variants; some older age ranges). Bias (DA–CA) demonstrated bidirectional behavior across studies: marked underestimation in some datasets (e.g., et al. Kwon: −0.80/−0.82; Lin: −0.82/−0.85; Pan: −0.07/−0.24) and clear overestimation in others (e.g., et al. Koç: +0.43/+0.34; Yavuz: +0.68/+0.68; Hato: +0.49/+0.76). Several papers also reported multiple Willems variants (e.g., Willems I vs. II; Willems SA vs. BC) with differing sex-specific performance, underscoring that the Willems approach is frequently reparameterized and not necessarily transportable without validation.

**Table 7:** Studies utilizing the Willems method for dental age assessment

Reference	Country/region	Sample size	MAE (M)	MAE (F)	MD DA-CA (M)	MD DA-CA (F)	Direction (M/F)
Alqerban, A. et al. 2021 [16]	Saudi Arabia (Saudi Arabian)	Total: 1146 Male: 605 Female: 541	1.37	1.37	-0.01	+ 0.06	None/Over
Bedek, I. et al. 2022 [18]	Croatia (Croatian population)	Total: 1996 Male: 1121 Female: 875	NR	NR	+ 0.41	+ 0.22	Over/Over
Brkic, H. et al. 2022 [20]	Croatia (Zagreb metropolitan area)	Total: 1492 Male: 704 Female: 788	0.78	0.70	-0.48 a	-0.48	Under/Under
Franco, A. et al. 2024 [25]	Brazil (mixed ancestry: Native Indians, Africans, Western Europeans)	Total: 5017 Male: 2443 Female: 2574	0.79	0.81	NR	NR	
Hato, E. et al. 2022 [29]	Turkey (Turkish children in the Central Black Sea Region)	Total: 400 Male: 196 Female: 204	NR	NR	+ 0.49	+ 0.76	Over/Over
Kar, M. et al. 2025 [31]	India (West Bengal) (Bengali)	Total: 411, Male: 204, Female: 207	NR	NR	NR	NR	
Koç, A. et al. 2021 [32]	Turkey (Eastern Turkish, various ethnic backgrounds)	Total: 919 Male: 460 Female: 459	0.79	0.81	+ 0.29	+ 0.34	Over/Over
Kwon, K. et al. 2023 [35]	China (Han population in Shanghai)	Total: 1259 Male: 634 Female: 625	0.88	0.99	-0.80	-0.82	Under/Under
Lin, Y. et al. 2022 [36]	China (Chinese Uyghur population)	Total: 831 Male: 354 Female: 477	1.16	1.17	-0.82	-0.85	Under/Under
Pan, J. et al. 2021 [39]	China (eastern China)	Total: 2367, Male: 1217, Female: 1150	0.70	0.79	-0.07	-0.24	Under/Under
Shan, W. et al. 2022 [42]	South China (population)	Total: 1,477 Male: 644 Female: 833	0.77	0.78	+ 0.09	-0.16	Over/Under
Sheriff, S. O et al. 2020 [43]	South India (South Indian children)	Total: 650 Male: 275 Female: 375	1.02	1.25	NR	NR	
Wang, J. et al. 2022 [45]	China (Eastern Chinese, Chinese Han population)	Total: 1211 Male: 582 Female: 629	0.95	1.00	-0.44	-0.09	Under/Under
Yavuz, B. S. et al. 2025 [46]	Turkey (Turkish children, northwestern Turkey)	Total: 1169 Male: 556 Female: 613	0.90*	1.01*	+ 0.68	+ 0.68	Over/Over
Şahin, T. N. et al. 2025 [40]	Turkey (Central Anatolia region, diverse ethnic backgrounds)	Total: 616 Male: 295 Female: 321	NR	NR	-0.43	-0.45	Under/Under

*NR *not reported

### London atlas method

The London Atlas method was reported in 6 study–method comparisons (Table 8). Sex-stratified MAE (when available) ranged from 0.58–1.19 years in males and 0.58–1.22 years in females (e.g., Kuppusamy; Lin; Dalben; Koç; Zhou). Reported bias (DA–CA) was generally small to moderate, with both slight underestimation (e.g., Lin: −0.06/−0.05; Koç: −0.07 in boys with + 0.28 in girls) and overestimation in other populations (e.g., Dalben: +0.60/+0.56; Yavuz: +0.05/+0.26; Kuppusamy: +0.01/+0.07). Two studies reported MAE without paired MD values (or vice versa), limiting cross-study comparability even within this single method.

**Table 8:** Studies utilizing the London Atlas method for dental age assessment

Reference	Country/region	Sample size	MAE (M)	MAE (F)	MD DA-CA (M)	MD DA-CA (F)	Direction (M/F)
Bakhsh, H. et al. 2024 [17]	Saudi Arabia (Saudi population)	Total: 711 Male: 357 Female: 354	NR	NR	+ 0.03	-0.00	Over/None
Dalben, E. et al. 2022 [21]	Brazil (Central-West Brazil)	Total: 1,104 Male: 595 Female: 509	1.19	1.22	+ 0.60	+ 0.56	Over/Over
Koç, A. et al. 2021 [32]	Turkey (Eastern Turkish, various ethnic backgrounds)	Total: 919 Male: 460 Female: 459	0.79	0.87	-0.07	+ 0.28	Under/Over
Kuppusamy, R. et al. 2025 [33]	Malaysia (multiethnic population)	Total: 523 Male: 279 Female: 244	0.58	0.60	+ 0.01 -0.002	+ 0.07 -0.08	None/Over Under/Under
Lin, Y. et al. 2022 [36]	China (Chinese Uyghur population)	Total: 831 Male: 354 Female: 477	0.83	0.88	-0.06	-0.05	Under/Under
Yavuz, S. et al. 2025 [46]	Turkey (Turkish children, northwestern Turkey)	Total: 1169 Male: 556 Female: 613	0.72*	0.74*	+ 0.05	+ 0.26	Over/Over
Zhou, J. et al. 2023 [47]	China (East China)	Total: 835, Male: 400, Female: 435	0.58	0.58	NR	NR	

*NR *not reported

### Other methods

Beyond the four most frequently reported approaches (Demirjian, Cameriere, Willems, London Atlas), the remaining dental age assessment (DAA) methods were represented by comparatively few study–method comparisons and were inconsistently reported (often lacking sex-stratified MAE and/or providing only mean differences [DA–CA]). This limited direct comparability across populations and precluded any meaningful synthesis beyond narrative comparison.

Across the included studies, Nolla most often underestimated chronological age, with mean differences ranging from near zero in some Turkish cohorts (e.g., ~ 0.0 to − 0.32 years) to substantial underestimation in Romanian and Saudi cohorts (approximately − 1.26 to − 1.71 years in Romania and − 1.07 to − 1.50 years in Saudi Arabia) [23, 32, 38]. When the MAE was reported, it was moderate (e.g., 0.84 years in boys and 0.94 years in girls in an Eastern Turkish cohort), but the direction and magnitude of bias appeared population- and age-structure dependent [32].

The evidence for the Haavikko method was sparse. In Croatian children, Haavikko showed relatively low MAE (~ 0.61 years) with a small overall underestimation (MD ≈ − 0.17 years) sex-stratified MD not always available. However, in another Croatian dataset, larger negative mean differences were reported (approximately − 0.6 years in boys and − 0.8 years in girls), again suggesting limited transportability without calibration [18].

Bedek-type models have been reported in limited settings [27]. In a South Indian cohort, MAE values were approximately 0.85 years (boys) and 0.88 years (girls) [43]. In an Eastern Turkish cohort, a Bedek 7-tooth model showed an MAE of ~ 0.70 years with a near-zero mean difference (≈ 0.0 in boys and − 0.1 in girls), although performance varied by the specific Bedek submodel (e.g., different tooth subsets suggested for boys vs. girls) [27].

## Discussion

This scoping review provides a contemporary map of panoramic radiograph–based dental age estimation methods used in children and adolescents between 2020 and 2025. At the method-family level, Willems-family methods were the most frequently reported and accounted for the largest proportion of assessed patients, followed by Demirjian-, Cameriere, and London Atlas-based approaches. Importantly, this pattern should be interpreted as a reflection of method adoption and reporting frequency rather than direct evidence of superior diagnostic performance. In addition, the high prevalence of the Willems family is not surprising because it is historically derived from the Demirjian framework and is widely used as a practical reparameterized alternative in both clinical and forensic settings. The frequency findings therefore indicate which methods are most commonly implemented in recent literature but not which method is universally the most accurate.

These findings differ from the previously prevailing view that the Demirjian method is the most commonly applied dental age estimation technique [11]. The Willems method employs a modified version of the Demirjian approach to assess radiographs by classifying tooth development into one of eight developmental stages. Each developmental stage of a given tooth is assigned a score based on sex-specific tables, and the scores for seven teeth are summed, with the total score directly corresponding to dental age [32]. The Willems method simplifies the Demirjian procedure by eliminating the additional conversion step. In essence, it represents a reconstructed version of the Demirjian method, in which the parameters of the original dental development model are adjusted through reregression [42].

The prominence of the Willems family in the included studies is consistent with its practical advantages and broad international uptake. However, the present results also show that Willems-based performance is population- and variant dependent. Across cohorts, the direction of bias (DA–CA) was bidirectional, with clear underestimation in some Chinese datasets and overestimation in several Turkish and Croatian cohorts. This variability was also observed in studies that used different Willems variants (e.g., Willems I vs II; SA vs BC), indicating that the Willems approach should not be treated as a single transportable model without local validation [45]. In practical terms, the method remains a useful and often accurate option, but its performance should be interpreted within the context of the population, age structure, and variant applied.

Studies conducted in East Chinese populations demonstrated that the Willems method provided greater accuracy in dental age estimation compared with the classical Demirjian method [39]. Similarly, research in South Chinese populations indicated that the Willems method was more appropriate than the Demirjian approach [43]. Dental age estimates obtained using the Willems method in Croatian populations were also significantly more accurate than those derived from the Demirjian method. Recent evidence further supports the accuracy and reliability of the radiographic Willems method [48]. Nevertheless, many authors publishing between 2020 and 2025 continued to combine the Willems method with other approaches to enhance precision [18, 29, 31, 32, 35, 36, 39, 40, 46].

The Demirjian method remains widely used and ranks as the second most frequently applied approach; however, numerous studies indicate that it is characterized by lower accuracy compared with alternative methods. In an Eastern Turkish population, the Nolla method demonstrated higher accuracy than the Demirjian method [18]. Similarly, studies in the Riyadh region of Saudi Arabia reported that the London Atlas method achieved greater accuracy than Demirjian, although the differences were not statistically significant. Owing to the need to improve its accuracy and validity, various modification strategies have been proposed. These include statistical approaches such as the polynomial curve fitting method (PCFM) [40], as well as integration with machine learning algorithms. Among these, Gradient Boosting and Random Forest models combining the Demirjian method with different machine learning techniques demonstrated the highest performance [49]. Convolutional neural network (CNN) models have also shown superior effectiveness in dental age estimation [50].

The Cameriere method, which ranks third in this review, is the only method in this comparison based on quantitative analysis of permanent tooth root development, measured by assessing the width of open root apices. One of its principal advantages is high precision, resulting from direct morphometric measurements and the use of regression equations to calculate dental age [27]. The method demonstrates good agreement between dental and chronological age, particularly in younger age groups [24]. Furthermore, it shows good reproducibility and reduced dependence on subjective observer interpretation. As with other methods, the effectiveness of the Cameriere method may be limited by population differences, emphasizing the need for population-specific formulas. Its applicability is reduced in older adolescents, in whom root mineralization is largely complete; consequently, differences in apical width become minimal, diminishing the method’s ability to discriminate age accurately [24, 26].

It should be noted that Professor Roberto Cameriere has authored numerous foundational and validation studies on open-apex-based dental age estimation [51–53]. The present review did not include Cameriere's original methodological publications as primary study entries, because those foundational papers predate the 2020–2025 search window and/or did not meet the minimum sample-size criterion (≥400 individuals). The specific Cameriere-family equations applied in each included study (European formula, population-specific modifications, Bayesian adaptations) are detailed in Tables 4 and 6.

The London Atlas method, ranked fourth, is based on comparison of panoramic radiographs with a reference atlas illustrating successive stages of dental development. Its main advantage lies in the comprehensive assessment of both deciduous and permanent dentition, enabling age estimation across a wide developmental range. The intuitive atlas-based format and the availability of dedicated software facilitate rapid application in clinical practice and reduce subjectivity [21, 47]. The accuracy of the London Atlas is particularly high in younger age groups but decreases during transitional periods, especially between late childhood and early adolescence. During this stage, the development of third molars plays a critical role; their high interindividual variability may increase the margin of error [34].

A comparison of MAE values across the four most frequently used methods suggests that the Cameriere method demonstrates the highest accuracy. However, as this parameter was not consistently reported across all studies and the analyzed groups varied in sample size, this finding should be interpreted with caution and regarded as indicative. Available evidence indicates that AI-based models, particularly convolutional neural networks (CNNs), demonstrated superior diagnostic performance with MAEs ranging from 0.03 to 0.7 years [54, 55].

Methods outside the four most frequently reported families (e.g., Nolla, Haavikko, Moorrees/MFH, GH, Nicodemo, Bedek, Chaillet, Blenkin-Evans) were represented by relatively few study–method comparisons and were often incompletely reported, particularly with respect to sex-stratified MAE and bias. This limited direct comparability and precluded strong conclusions. Nevertheless, the available evidence suggests that several of these methods may perform well in specific populations, whereas others show substantial bias without local calibration. This pattern again supports the broader conclusion of the review: method performance is population-dependent [55, 56], and even historically established methods may require regional validation before clinical or forensic use.

Several limitations of the evidence base should be considered when interpreting these findings. First, most included studies were retrospective and clinic-based, which may introduce selection bias and limit generalizability to the broader pediatric population. Second, verification of chronological age was not uniformly reported; although many studies used official records, others provided limited or no information on reference standard verification. Third, reporting of blinding and reproducibility procedures was inconsistent, and not all studies provided both interrater and intrarater reliability. Fourth, despite the large overall number of participants, age distributions within individual studies were often uneven, which may affect performance estimates in specific age strata. Finally, although risk of bias was assessed using an adapted Newcastle–Ottawa Scale, the appraisal was intended to support descriptive interpretation rather than to exclude studies or generate pooled estimates. Therefore, the findings should be interpreted as a structured map of current evidence rather than as a quantitative synthesis of diagnostic accuracy.

## Conclusion

This scoping review demonstrates that panoramic radiograph-based dental age estimation methods are applied heterogeneously across populations, with the Willems family of methods being the most frequently reported in recent literature. Because no single method has proven universally transferable, clinicians should not assume that validation in one population justifies direct application to a different ethnic or geographic group. In settings where panoramic radiography is routinely performed, method selection should be guided by the best available local validation evidence; where no population-specific data exist, practitioners are advised to apply two or more methods in parallel and interpret results with an appropriate margin of uncertainty. The directional bias values (DA–CA) reported across studies reflect systematic tendencies of specific methods in specific populations rather than universal properties of those methods; these findings should not be over-generalized. The magnitude and direction of bias varied substantially with population composition, age structure, and the particular variant applied — reinforcing that such conclusions must remain population-specific. Taken together, these observations point to a priority for future research: population-specific validation studies paired with standardized reporting of MAE, bias (DA–CA), sex-stratified outcomes, and reliability measures. 

## Supplementary Information

Supplementary Material 1.

Supplementary Material 2.

Supplementary Material 3.

## Data Availability

All data generated or analysed during this study are included in this published article.
