# Supplementary material for: Panoramic radiograph–based dental age estimation in children and adolescents using multi-tooth methods: a scoping review of methods used and reported accuracy (2020–2025)
Source: BMC Oral Health. 2026 Jul 15;26:2005. doi: 10.1186/s12903-026-09263-w (PMC13663167; doi:10.1186/s12903-026-09263-w)
Supplement: Supplementary file 2 [file 12903_2026_9263_MOESM1_ESM.docx]

**Supplementary Table 2.** Quality assessment of included studies The Newcastle-Ottawa Scale (NOS).

| Author  (Year) | Clareness of stated aim  (0-2) | Sample selection | | | | Comparability | | Outcome | | NOS score  (0-16) | Quality  (low/  moderate/high |
| --- | --- | --- | --- | --- | --- | --- | --- | --- | --- | --- | --- |
|  |  | Sample representativeness  (0-2) | Sample size  (0-2) | Non-respondents  (0-2) | Exposure Assessment  (0-2) | Control of confounding factors  (0-1) | Comparability of participants  (0-1) | Assessment of the outcome  (0-2) | Statistical tests  (0-2) |  |  |
| AlOtaibi, N. et al. 2023 [16] | 2 | 0 | 1 | 2 | 2 | 0 | 1 | 2 | 1 | 11 | moderate |
| AlOtaibi, N. et al. 2023 [17] | 2 | 0 | 1 | 2 | 2 | 0 | 1 | 2 | 1 | 11 | moderate |
| Alqerban, A. et al. 2021 [18] | 2 | 0 | 2 | 2 | 2 | 1 | 0 | 2 | 2 | 13 | high |
| Bakhsh, H. et al. 2024 [19] | 2 | 1 | 1 | 2 | 2 | 0 | 1 | 2 | 1 | 12 | moderate |
| Bedek, I. et al. 2022 [20] | 2 | 0 | 2 | 2 | 2 | 0 | 0 | 2 | 1 | 11 | moderate |
| Briem Stamm, A. et al. 2022 [21] | 1 | 0 | 1 | 2 | 2 | 0 | 0 | 2 | 1 | 9 | moderate |
| Brkic, H. et al. 2022 [22] | 2 | 0 | 2 | 2 | 2 | 0 | 0 | 2 | 2 | 12 | moderate |
| Dalben, E et al. 2022 [23] | 2 | 0 | 2 | 2 | 2 | 1 | 0 | 2 | 2 | 13 | high |
| Darici, Aet al. 2024 [24] | 2 | 1 | 1 | 2 | 2 | 0 | 1 | 2 | 1 | 12 | moderate |
| Duruk, Get al. 2022 [25] | 2 | 0 | 2 | 2 | 2 | 1 | 1 | 2 | 1 | 13 | high |
| El-Desouky, S. Set al. 2023 [26] | 2 | 0 | 1 | 2 | 2 | 1 | 0 | 2 | 1 | 11 | moderate |
| Franco, Aet al. 2024 [27] | 2 | 0 | 2 | 2 | 2 | 1 | 1 | 2 | 1 | 13 | high |
| Goncalves do Nascimento, L. et al. 2020 [28] | 1 | 1 | 1 | 2 | 2 | 0 | 0 | 2 | 1 | 10 | moderate |
| Gümüşboğa Z. S et al. 2024 [29] | 2 | 0 | 2 | 2 | 2 | 0 | 1 | 2 | 1 | 12 | moderate |
| Gupta, S. et al. 2023 [30] | 2 | 1 | 1 | 2 | 2 | 1 | 0 | 2 | 1 | 12 | moderate |
| Hato, E. et al. 2022 [31] | 2 | 0 | 1 | 2 | 2 | 0 | 1 | 2 | 1 | 11 | moderate |
| Ishwarkumar, S. et al. 2022 [32] | 2 | 0 | 1 | 2 | 2 | 1 | 1 | 2 | 1 | 12 | moderate |
| Kar, M. et al. 2025 [33] | 2 | 0 | 1 | 2 | 2 | 1 | 1 | 2 | 1 | 12 | moderate |
| Koc, A et al. 2021 [34] | 2 | 0 | 1 | 2 | 2 | 1 | 1 | 2 | 2 | 13 | high |
| Kuppusamy R. et al. 2025 [35] | 2 | 0 | 1 | 2 | 2 | 0 | 0 | 2 | 2 | 11 | moderate |
| Kuremoto, Ket al. 2022 [36] | 2 | 0 | 2 | 2 | 2 | 0 | 1 | 2 | 1 | 12 | moderate |
| Kwon, K. et al. 2023 [37] | 2 | 1 | 1 | 2 | 2 | 0 | 1 | 2 | 2 | 13 | high |
| Lin, Y. et al. 2022 [38] | 2 | 1 | 1 | 2 | 2 | 0 | 0 | 2 | 2 | 12 | moderate |
| Moca, A. E. et al. 2022 [39] | 2 | 0 | 1 | 2 | 2 | 0 | 0 | 2 | 1 | 10 | moderate |
| Moga, L. et al. 2025 [40] | 2 | 0 | 1 | 2 | 2 | 0 | 0 | 2 | 1 | 10 | moderate |
| Pan, J et al. 2021 [41] | 2 | 0 | 2 | 2 | 2 | 0 | 1 | 2 | 2 | 13 | high |
| Sahin, T. N. et al. 2025 [42] | 2 | 0 | 1 | 2 | 2 | 0 | 1 | 2 | 1 | 11 | moderate |
| Sgheiza, V. et al. 2023 [43] | 2 | 0 | 2 | 2 | 2 | 0 | 0 | 2 | 1 | 11 | moderate |
| Shan, W. et al. 2022 [44] | 2 | 0 | 2 | 2 | 2 | 0 | 0 | 2 | 2 | 12 | moderate |
| Sheriff, S. O. et al. 2020 [45] | 2 | 0 | 1 | 2 | 2 | 0 | 0 | 2 | 1 | 10 | moderate |
| Valente, R. et al. 2024 [46] | 2 | 0 | 2 | 2 | 2 | 0 | 1 | 2 | 1 | 12 | moderate |
| Wang, J. et al. 2022 [47] | 2 | 1 | 2 | 2 | 2 | 0 | 0 | 2 | 1 | 12 | moderate |
| Yavuz, B. et al. 2025 [48] | 2 | 0 | 2 | 2 | 2 | 0 | 0 | 2 | 2 | 12 | moderate |
| Zhou J. et al. 2023 [49] | 2 | 0 | 1 | 2 | 2 | 0 | 0 | 2 | 1 | 10 | moderate |

Total score: 13–16 points (high quality and low risk of bias), 9–12 points (moderate quality and moderate risk of bias); <9 points (low quality and high risk of bias).
