# Supplementary material for: Panoramic radiograph–based dental age estimation in children and adolescents using multi-tooth methods: a scoping review of methods used and reported accuracy (2020–2025)
Source: BMC Oral Health. 2026 Jul 15;26:2005. doi: 10.1186/s12903-026-09263-w (PMC13663167; doi:10.1186/s12903-026-09263-w)
Supplement: Supplementary file 3 [file 12903_2026_9263_MOESM3_ESM.docx]

1. Abuabara A, Do Nascimento T, Trentini SM, Costa Gonçalves AM, Hueb De Menezes-Oliveira MA, Madalena IR, et al. Evaluating the accuracy of generative artificial intelligence models in dental age estimation based on the Demirjian’s method. Front Dent Med. 2025 Jul 29;6:1634006. doi:10.3389/fdmed.2025.1634006
2. Alrashidi M, Al-Moshiqah A, Kolarkodi SH, Alotaiby F. A Comparative Study of the Assessment of the Accuracy of Willems’ and Demirjian’s Methods in Dental Age Estimation of the Saudi Arabian Population. Cureus. 2023 Dec 7;15(12):e50128 doi:10.7759/cureus.50128
3. Balel Y, Sağtaş K, Bülbül HN. Development and evaluation of a deep learning-based system for dental age estimation using the demirjian method on panoramic radiographs. BMC Oral Health. 2025 Jul 16;25(1):1172. doi:10.1186/s12903-025-06420-5
4. Cadenas de Llano-Pérula M, Kihara E, Thevissen P, Nyamunga D, Fieuws S, Kanini M, et al. Validating dental age estimation in Kenyan black children and adolescents using the Willems method. Med Sci Law. 2021 Jul;61(3):180–5. doi:10.1177/0025802420977379 PubMed PMID: 33270504.
5. Caldas IM, Cardoso HFV. Socioeconomic differences in permanent teeth mineralization of Portuguese girls and boys from Porto, Portugal. Anthropol Anz Ber Uber Biol-Anthropol Lit. 2021 Nov 18;78(4):267–77. doi:10.1127/anthranz/2021/1313 PubMed PMID: 33595590.
6. Chandail K, Goyal V, Kaul M, Dutt S, Koul T, Misgar BA. Estimation of dental age using Willems method and comparing it with Demirjian’s method in 7-14-year-old children of Uttarakhand. J Indian Soc Pedod Prev Dent. 2022 Mar;40(1):43–7. doi:10.4103/jisppd.jisppd_333_21 PubMed PMID: 35439882.
7. Chaudhry K, Talwar M, Choudhary A, Patnana AK, Vanga NR, Lehl GK. A Comparative Evaluation of Three Different Dental Age Estimation Methods in India: A Test of Ethnic Variability. Int J Clin Pediatr Dent. 2020 Feb;13(1):16–20. doi:10.5005/jp-journals-10005-1708
8. Cherian JM, Thomas AM, Kapoor S, Kumar R. Dental age estimation using Willems method: A cross-sectional study on children in a North Indian city. J Oral Maxillofac Pathol. 2020 May;24(2):383–8. doi:10.4103/jomfp.JOMFP_299_19
9. Choi TM, Kramer GJC, Goos JAC, Mathijssen IMJ, Wolvius EB, Ongkosuwito EM. Evaluation of dental maturity in Muenke syndrome, Saethre-Chotzen syndrome, and TCF12-related craniosynostosis. Eur J Orthod. 2022 May 24;44(3):287–93.

doi:10.1093/ejo/cjab056 PubMed PMID: 34424951; PubMed Central PMCID: PMC9127722.

10. Collier SL, Lewis JM, Kasper KA, Marks MK, Heidel RE. Dental Age Assessment of United States Black and White Children: Performance Reliability of Harris and McKee (1990). Am J Forensic Med Pathol. 2023 Mar 1;44(1):33–41. doi:10.1097/PAF.0000000000000797 PubMed PMID: 36165591.
11. Dogan OB, Boyacioglu H, Goksuluk D. Machine learning assessment of dental age classification based on cone-beam CT images: a different approach. Dento Maxillo Facial Radiol. 2024 Jan 11;53(1):67–73. doi:10.1093/dmfr/twad009 PubMed PMID: 38214945; PubMed Central PMCID: PMC11003658.
12. Guo YX, Bu WQ, Tang Y, Wu D, Yang H, Meng HT, et al. Dental Age Estimation in Northern Chinese Han Children and Adolescents Using Demirjian’s Method Combined with Machine Learning Algorithms. Fa Yi Xue Za Zhi. 2024 Apr 25;40(2):135–42. doi:10.12116/j.issn.1004-5619.2023.231208 PubMed PMID: 38847027.
13. Guo, YX., Han, M., Chi, Y. et al. Accurate age classification using manual method and deep convolutional neural network based on orthopantomogram images. Int J Legal Med 2021;135:1589–1597. https://doi.org/10.1007/s00414-021-02542-x
14. Jayaraman J, Mendez MJC, Gakunga PT, Roberts G. Age estimation of Hispanic children in the United States: Development and validation of dental reference dataset based on two staging systems. Leg Med Tokyo Jpn. 2022 May;56:102033. doi:10.1016/j.legalmed.2022.102033 PubMed PMID: 35151981.
15. Kahm SH, Kim JY, Yoo S, Bae SM, Kang JE, Lee SH. Application of entire dental panorama image data in artificial intelligence model for age estimation. BMC Oral Health. 2023 Dec 15;23(1):1007. doi:10.1186/s12903-023-03745-x
16. Kim S, Lee YH, Noh YK, Park FC, Auh QS. Age-group determination of living individuals using first molar images based on artificial intelligence. Sci Rep. 2021 Jan 13;11(1):1073. doi:10.1038/s41598-020-80182-8 PubMed PMID: 33441753; PubMed Central PMCID: PMC7806774.
17. Kolcakoglu K, Amuk M, Kizilci E, Sirin Sarıbal G, Askaroglu Ö, Korkut Dİ. Chronological age estimation by measuring pulp chamber volume in teeth with open apices: A CBCT analysis. Dent Med Probl. 2025 Sep-Oct;62(5):899-906. doi: 10.17219/dmp/153419. PMID: 40485625.
18. Kurniawan A, Saelung M, Rizky BN, Chusida A, Prakoeswa BFWR, Nefertari G, Pradue AF, Margaretha MS, Alias A, Marya A. Dental age estimation using a convolutional neural network algorithm on panoramic radiographs: A pilot study in Indonesia. Imaging Sci Dent. 2025 Mar;55(1):28-36. doi: 10.5624/isd.20240134. Epub 2025 Mar 10. PMID: 40191398; PMCID: PMC11966015.
19. Lashin HI, Sharif AF, Ghaly MS, El-Desouky SS, Elhawary AE. Bridging gaps in age estimation: a cross-sectional comparative study of skeletal maturation using Fishman method and dental development using Nolla method among Egyptians. Int J Legal Med. 2025 Mar;139(2):695–714. doi:10.1007/s00414-024-03394-x PubMed PMID: 39760867; PubMed Central PMCID: PMC11850478.
20. Lee SS, Kumagai A. Comparison of the accuracy and bias of Demirjian’s method for East Asian children and development of Japanese population-specific age estimation formulae. Leg Med Tokyo Jpn. 2024 Nov;71:102529. doi:10.1016/j.legalmed.2024.102529 PubMed PMID: 39293287.
21. Lee SS, Kumagai A. The applicability of Willems’ and Lee’s dental age estimation methods for Japanese children and the comparison with the Korean population. Leg Med Tokyo Jpn. 2022 Sep;58:102094. doi:10.1016/j.legalmed.2022.102094 PubMed PMID: 35688110.
22. Li Z, Xiao N, Nan X, Chen K, Zhao Y, Wang S, Guo X, Gao C. Automatic dental age estimation in adolescents via oral panoramic imaging. Front Dent Med. 2025 Jun 26;6:1618246. doi: 10.3389/fdmed.2025.1618246. PMID: 40642202; PMCID: PMC12241049.
23. Machado MVF, Soares MQS, Baz AMSA, Junqueira JLC, Franco A. A large sample-sized study on dental development of children treated at the Central Dental Clinic (OCEx) of the Brazilian Army. Clin Oral Investig. 2022 Aug;26(8):5439-5447. doi: 10.1007/s00784-022-04511-z. Epub 2022 Apr 29. PMID: 35484357.
24. Mazloom Nezhad SM, Mohd Faizal Abdullah ER, Ibrahim N, Bakhsh HH, Ishtiaq U, Rahmat RAA, et al. A hybrid convolutional neural network model for dental age estimation using buccal alveolar bone level for Saudi children. BMC Oral Health. 2025 Aug 16;25(1):1331. doi:10.1186/s12903-025-06694-9
25. Ong SH, Chang I, Kim H, Song JS, Shin TJ, Hyun HK, et al. Secular changes in dental development of Korean children aged 4 to 16 years over a 10-year period. J Clin Pediatr Dent. 2024 Jul;48(4):68–73. doi:10.22514/jocpd.2024.080 PubMed PMID: 39087216.
26. Ong SH, Kim H, Song JS, Shin TJ, Hyun HK, Jang KT, et al. Fully automated deep learning approach to dental development assessment in panoramic radiographs. BMC Oral Health. 2024 Apr 6;24(1):426. doi:10.1186/s12903-024-04160-6 PubMed PMID: 38582843; PubMed Central PMCID: PMC10998373.
27. Paz Cortés MM, Rojo R, Alía García E, Mourelle Martínez MR. Accuracy assessment of dental age estimation with the Willems, Demirjian and Nolla methods in Spanish children: Comparative cross-sectional study. BMC Pediatr. 2020 Dec;20(1):361. doi:10.1186/s12887-020-02247-x
28. Peker RB. Artificial intelligence versus human expertise: reliability of ChatGPT and the London atlas for dental age estimation using panoramic radiographs. BMC Oral Health. 2025 Nov 28;25(1):2002. doi:10.1186/s12903-025-07360-w
29. Pliska B, Nahvi A, Pakdaman N, Dadgar S, Aryana M, Sobouti F. Radiological Evaluation of the Accuracy of Demirjian, Nolla, and Willems Methods for Dental Age Estimation in 3-17-Year-Old Iranian Children. BioMed Res Int. 2024 Jul 3;2024:8783660. doi:10.1155/2024/8783660 PubMed PMID: 38988904; PubMed Central PMCID: PMC11236468.
30. Polat Y, Çelenk S. A comparison of estimated age based on pulp volume from cone beam computed tomography (CT) images and panoramic radiography data with chronological age. J Clin Pediatr Dent. 2024 Mar;48(2):149-162. doi: 10.22514/jocpd.2024.043. Epub 2024 Mar 3. PMID: 38548645.
31. Prakoeswa BFWR, Kurniawan A, Chusida A, Marini MI, Rizky BN, Margaretha MS, Utomo H, I'zaaz Darmawan A, Aisyah AKN, Alias A, Wahjuni OR, Marya A. Children and Adolescent Dental Age Estimation by the Willems and Al Qahtani Methods in Surabaya, Indonesia. Biomed Res Int. 2022 Jul 7;2022:9692214. doi: 10.1155/2022/9692214. PMID: 35845954; PMCID: PMC9283048.
32. Rezende Machado AL, Borges BS, Cameriere R, Palhares Machado CE, Alves da Silva RE. Evaluation of Cameriere and Willems age estimation methods in panoramic radiographs of Brazilian children. J Forensic Odontostomatol. 2020 Dec 30;38(3):8-15. PMID: 33507162; PMCID: PMC8565658.
33. Shi L, Zhou Y, Lu T, Fan F, Zhu L, Suo Y, Chen Y, Deng Z. Dental age estimation of Tibetan children and adolescents: Comparison of Demirjian, Willems methods and a newly modified Demirjian method. Leg Med (Tokyo). 2022;55:102013. doi: 10.1016/j.legalmed.2022.102013. PMID: 34999531.
34. da Silveira Sousa AM, Jacometti V, Sato CM, AlQahtani S, da Silva RHA. Technical note: RP Atlas-an unprecedented proposal for a Brazilian population dental development chart based on the London atlas. Am J Biol Anthropol. 2024 Aug;184(4):e24906. doi:10.1002/ajpa.24906 PubMed PMID: 38294146.
35. Sivri MB, Taheri S, Kırzıoğlu Ercan RG, Yağcı Ü, Golrizkhatami Z. Dental age estimation: A comparative study of convolutional neural network and Demirjian's method. J Forensic Leg Med. 2024 Apr;103:102679. doi: 10.1016/j.jflm.2024.102679. PMID: 38537363.
36. Sributsayakarn N, Intharah T, Hirunchavarod N, Pornprasertsuk-Damrongsri S, Jirarattanasopha V. Automated Age and Sex Estimation From Dental Panoramic Radiographs. Int Dent J. 2025 Dec;75(6):103967. doi:10.1016/j.identj.2025.103967
37. Sun KT, Wu YZ, Hsu JT, Huang HL. Correlation between dental age and chronological age of Taiwanese children on panoramic X-ray images by different evaluation methods. J Chin Med Assoc. 2025 Feb 1;88(2):108-115. doi: 10.1097/JCMA.0000000000001182. Epub 2024 Oct 22. PMID: 39434210; PMCID: PMC12719002.
38. Sun KT, Wu YZ, Hsu JT, Huang HL. Influence of sex and timing of mixed dentition on discrepancies between chronological age and dental age in Taiwanese children. J Dent Sci. 2024 Oct;19(4):1942-1950. doi: 10.1016/j.jds.2024.08.003. Epub 2024 Aug 17. PMID: 39347033; PMCID: PMC11437346.
39. Tyagi A, Srivastava N, Rana V, Kaushik N, Goel S, Khera AK. Accuracy of Different Dental Age Estimation Methods Using Cone Beam Computed Tomography: A Comparative Study. Int J Clin Pediatr Dent. 2024 May;17(5):558-564. doi: 10.5005/jp-journals-10005-2860. PMID: 39355178; PMCID: PMC11440676.
40. Willmann C, Fernandez De Grado G, Kolb C, Raul JS, Musset AM, Gros CI, et al. Accuracy of Age Estimation Using Three Dental Age Estimation Methods in a Young, Large, and Multiethnic Patient Sample. Dent J. 2023 Dec 13;11(12):288. doi:10.3390/dj11120288
41. Wang J, Fan L, Shen S, Sui M, Zhou J, Yuan X, et al. Comparative assessment of the Willems dental age estimation methods: a Chinese population-based radiographic study. BMC Oral Health. 2022 Sep 3;22(1):373. doi:10.1186/s12903-022-02418-5 PubMed PMID: 36057573; PubMed Central PMCID: PMC9440589.
42. Wang J, Dou J, Han J, Li G, Tao J. A population-based study to assess two convolutional neural networks for dental age estimation. BMC Oral Health. 2023 Feb 17;23(1):109. doi:10.1186/s12903-023-02817-2
43. Wen D, Ding Z. Comparing the accuracy of Demirjian and Nolla methods and establishing a new method for dental age estimation in northeastern Chinese children. 2023 Feb 12;7(4):685-693. doi: 10.1080/20961790.2021.2024655. PMID: 36817253; PMCID: PMC9930805.
44. Yousefi F, Mohammadi Y, Ahmadvand M, Razaghi P. Dental age estimation using cone-beam computed tomography: A systematic review and meta-analysis. Imaging Sci Dent. 2023 Jun;53(2):91–9. doi:10.5624/isd.20221226 PubMed PMID: 37405204; PubMed Central PMCID: PMC10315230.
